# Linking the Scores of the Montreal Cognitive Assessment 5-min and the interRAI Cognitive Performance Scale in Older Adults With Mild Physical or Cognitive Impairment

**DOI:** 10.3389/fpsyt.2021.705188

**Published:** 2021-09-14

**Authors:** Björn Andersson, Hao Luo, Gloria H. Y. Wong, Terry Y. S. Lum

**Affiliations:** ^1^Centre for Educational Measurement, University of Oslo, Oslo, Norway; ^2^Department of Social Work and Social Administration, The University of Hong Kong, Hong Kong, SAR China

**Keywords:** crosswalk, cognitive screening, MoCA 5-min, cognitive performance scale, test equating

## Abstract

**Background:** Bridging scores generated from different cognitive assessment tools is necessary to efficiently track changes in cognition across the continuum of care. This study linked scores from the Montreal Cognitive Assessment-5 min (MoCA 5-min) to the interRAI cognitive Performance Scale (CPS), commonly adopted tools in clinical and long-term care settings, respectively.

**Methods:** We included individual-level data from persons who participated in a home- and community-based care program for older people with mild impairment in Hong Kong. The program used the interRAI-Check Up instrument for needs assessment and service matching between 2017 and 2020. Each participant's cognitive performance was assessed using CPS, CPS Version 2 (CPS2), and MoCA 5-min. We performed equipercentile linking with bivariate log-linear smoothing to establish equivalent scores between the two scales.

**Results:** 3,543 participants had valid data on both scales; 66% were female and their average age was 78.9 years (SD = 8.2). The mean scores for MoCA 5-min, CPS, and CPS2 were 18.5 (SD = 5.9), 0.7 (SD = 0.7), and 1.3 (SD = 1.1), respectively. A CPS or CPS2 score of 0 (intact cognition) corresponds to MoCA 5-min scores of 24 and 25, respectively. At the higher end, a CPS score of 3 (moderately impaired) and a CPS2 score of 5 (moderately impaired Level-2) corresponded to MoCA 5-min scores of 0 and 1, respectively. The linking functions revealed the floor and ceiling effects that exist for the different scales, with CPS and CPS2 measuring more-severe cognitive impairment while the MoCA 5-min was better suited to measure mild impairment.

**Conclusions:** We provided score conversions between MoCA 5-min and CPS/CPS2 within a large cohort of Hong Kong older adults with mild physical or cognitive impairment. This enabled continuity in repeated assessment with different tools and improved comparability of cognitive scores generated from different tools from diverse populations and research cohorts.

## Introduction

Assessing cognition in the aging population is necessary to understand the magnitude of loss in cognitive performance. In the long-term care system, the Cognitive Performance Scale (CPS), originally developed using data from the Minimum Data Set (MDS) assessment (1), is a widely used tool. CPS items were designed to assess the person's actual performance in remembering, thinking coherently, and organizing daily self-care activities as these are considered potentially crucial threats to personal independence and increase the risk for long-term care facility admission (2). The scale has been implemented in the MDS and interRAI assessment instruments that routinely collect data on vulnerable persons' clinical and functional status to improve their quality of life (3). In addition to providing a descriptive foundation of a person's cognition, CPS scores are used for triggering the cognitive loss Clinical Assessment Protocol (CAP) in the interRAI system. The interRAI system includes several CAPs designed to inform and guide care and service planning. Specifically, the cognitive loss CAP focuses on helping persons with intact cognitive ability or mild cognitive impairment to remain as independent as possible for as long as possible (4). The interRAI instruments have been widely adopted by home care and long-term care facilities, with assessments administered to over 50 million people worldwide (5–7). The CPS is also embedded in the Resident Assessment Instrument – Mental Health (RAI-MH), a valid screening measure of cognitive performance among adult psychiatric inpatients (8). In addition, the CPS has been extended to a new CPS Version 2 (CPS2) to improve its sensitivity to early cognitive impairment (9).

In the general health care environment, the Montreal Cognitive Assessment (MoCA) is a commonly used tool for screening cognitive impairment and dementia (10). The MoCA was designed to facilitate early and accurate detection of mild cognitive impairment by front-line physicians. It assesses multiple cognitive domains (including visuospatial/executive, naming, memory, attention, language, abstraction, delayed recall, and orientation) and was originally developed as a paper-and-pencil tool with 30 questions requiring the physical presence of the examinee and takes 10–20 min to administer. The validity of MoCA (including content, construct, and criteria validity) has been evaluated by studies of different populations and with different modeling frameworks. Although considerable variability in the sensitivity, specificity, and psychometric properties of the MoCA has been observed across populations with different characteristics, it demonstrates overall satisfactory performance in detecting mild cognitive impairment and dementia (11). Previous evidence also suggests that, compared to the Mini-Mental State Examination (MMSE), MoCA demonstrates superiority in detecting more subtle changes in cognition that may signal mild cognitive impairment caused by many illnesses (including Alzheimer's disease, Parkinson's disease, and stroke) (12). However, as reported by previous studies, the MoCA has a prominent floor effect and may not be suitable for measuring cognitive ability in people with severe impairment (e.g., those with MMSE scores of 10 or below) (13–15).

More recently, shorter versions of the MoCA have been developed to address the limited time available for cognitive assessment in many clinical settings (16–19). The MoCA 5-min protocol (MoCA 5-min), based on the Hong Kong version of the MoCA, has been developed as a very brief cognitive screening tool administered at the bedside or over the telephone to accommodate challenging face-to-face assessment situations (19). The MoCA 5-min has been validated in patients with stroke and transient ischemic attack (TIA), and score conversion between the MoCA 5-min and the full version of MoCA has been reported (15, 19). Owing to its shorter administration time, the MoCA 5-min has gained popularity recently in locations as diverse as Hong Kong, France, and Tanzania (20–22).

Older persons may move between care settings (e.g., home care, long-term care facilities, hospitals, and rehabilitation) as the levels of care they need change (3). Although cognitive assessments are often routinely carried out as part of the comprehensive assessment within the same setting, a person's cognitive status before entering the setting is often unknown. In situations where scores of previous cognitive assessments are available, establishing a valid trajectory of cognitive function remains difficult as the scores are unlikely to be from the same assessment tool. Consequently, determining any change in a person's cognitive status is particularly challenging if the person is newly admitted to the facility and no benchmark cognitive score is available. Linking the scores of different cognitive assessment tools allows continuous tracking of cognitive performance across the continuum of care, leverages existing records, and reduces the assessment burden. This also enables the identification of homogeneous groups of individuals with similar levels of cognitive impairment from different populations, which in turn allows further contextual-level enquiries. This study aimed to bridge scores from the MoCA 5-min to the CPS and CPS2 using assessment data from a large cohort of older adults in Hong Kong.

## Method

### Sample

We used baseline assessments of participants in a home and community-based care program for older people with mild impairment in Hong Kong. The program uses the Hong Kong Chinese version of the interRAI-Check Up (interRAI-CU) instrument for needs assessment and service matching (23). Additionally, participants' cognitive ability was assessed using the MoCA 5-min. All assessors received 2-day training and were accredited by the Social Welfare Department of the Hong Kong Government. Data collection was conducted between April 2017 and September 2020. Participants were assessed when they joined the program (the baseline assessment) for eligibility and service matching and were (and will be continuously) reassessed annually for care planning. All participants provided informed written consent. Ethical approval was obtained from the Review Board of the Human Research Ethics Committee for Non-Clinical Faculties at the University of Hong Kong (EA1709028).

### Instruments and Measures

This interRAI-CU was recommended for use with two specific subgroups: (1) persons who could perform instrumental activities without the help of others, and (2) persons who required the help of others with meal preparation or housework only. The foundation reference of the interRAI-CU is the interRAI Home Care (HC), an assessment tool for persons in the community receiving home care services. The interRAI-CU is a shorter tool with about 100 items developed to support programs that address the needs of persons living independently in the community. It includes assessor-ratings on multiple domains, including cognition and communication, mood and psychosocial well-being, functional status, and health condition.

#### CPS

The CPS was generated using four interRAI items: short-term memory, cognitive decision-making, making oneself understood by others, and dependence in eating (1). Short-term memory was assessed by a binary item indicating whether the person could recall three unrelated items after 5 min. The cognitive decision-making item measures the person's cognitive skill for making decisions regarding daily living tasks. The person's cognitive skills were rated as 0 for independent, 1 for modified independence, 2 for minimally impaired, 3 for moderately impaired, 4 for severely impaired, and 5 for no discernible consciousness. Making oneself understood by others measured the person's ability to express information content (verbal and non-verbal). Expression ability was rated from 0 for understood (expresses ideas without difficulty) to 4 for rarely or never understood. The dependence in eating item was rated from 0 for independent to 6 for total dependence and was intended to anchor the most cognitively impaired category. Accredited assessors scored all items.

The total CPS score ranges from 0 for “cognitively intact” to 6 for “severe impairment,” and is calculated according to a hierarchical structure designed to replicate the progressive nature of cognitive decline (9). The tool correlates substantially with the MMSE and other scales such as the MDS-cognition Scale, the Psychogeriatric Dependency Rating Scale (PGDRS), and the Activities of Daily Living Scale (ADL) in nursing home residents (1, 24) and people receiving home care services (25).

#### CPS2

CPS2 was developed to detect changes more sensitively in earlier stages of cognitive decline (9). It is based on six interRAI items: capacity to manage finances, capacity to manage medications, short-term memory, making oneself understood by others, decision-making, and walking. The managing finances and medications items measure the person's presumed ability to handle bills, credit cards, and household expenses and medication, respectively, both with ratings ranging from 0 for independent to 6 for total dependence. They are also standard items for assessing the person's ability in Instrumental Activities of Daily Living (IADL). The dependence in eating item in CPS was substituted by dependency level in walking in CPS2. The total score of CPS2 ranges from 0 for “cognitively intact Level one” to 8 for “very severe impairment.” A previous study demonstrated a significant correlation between individual CPS2 items and MMSE, with correlation coefficients ranging from −0.44 (managing finances) to −0.69 (decision making). The total CPS2 score was highly correlated with CPS (*r* = 0.93), MMSE (−0.76), and external measures of dementia diagnosis, function, living status, and distress (9).

#### MoCA 5-min

The MoCA 5-min consists of four sub-tests extracted from the MoCA examining four cognitive domains: attention, verbal learning and memory, executive functions/language, and orientation (19). The attention domain is assessed by the immediate recall of five words, with scores ranging from 0 to 5 (1 point for each word correctly recalled). The executive functions/language domain is assessed by a 1-min verbal fluency test with scores ranging from 0 to 9. Orientation is measured by six items on data and geographic orientation, with 1 point for each correct answer. Memory is tested by delayed recall and recognition of five words learned in the first task (immediate recall), with scores ranging from 0 to 10. The total scores of the MoCA 5-min range between 0 and 30. Previous evidence suggested a high correlation between the MoCA 5-min protocol and the MoCA (*r* = 0.87). It also performed well in differentiating people with and without cognitive impairment in people with stroke or TIA (19).

Other measures obtained from interRAI-CU include age, sex, educational level, the ADL - Hierarchy Scale (ADL-H), and dementia diagnosis. The ADL-H is a summary scale measuring a person's functional status, with total scores ranging from 0 (independent) to 6 (total dependence). Earlier research suggested that individual ADL-H items can be classified into early loss, middle loss, and later loss components and a significant association between the scale score and external ADL criteria such as the time involved in formal and informal care (26, 27). Dementia diagnosis was measured by the diagnosis of Alzheimer's disease, other dementia, or dementia of unknown origin.

### Statistical Analysis

The relationships between CPS, CPS2, and MoCA 5-min were initially assessed using Pearson correlations. As our sample was limited to older persons with mild physical or cognitive impairment, higher CPS values (i.e., severe cognitive impairment) may not be observed. A ceiling effect of CPS and CPS2 might be expected that may subsequently lead to a biased estimate of the Pearson correlation coefficient. Therefore, we further evaluated the association between CPS/CPS2 and MoCA 5-min using Kruskal-Wallis rank sum tests, a non-parametric method for testing the association between two variables (28). For each CPS and CPS2 score, we also compared and tested the mean age and ADL-H scores using Kruskal-Wallis rank sum tests. We fitted three simple logistic regression models to assess the predictive accuracy of the CPS, CPS2, and MoCA 5-min for detecting dementia. It is important to note that this predictive accuracy evaluation is mainly exploratory as the dementia diagnostic rate is very low in Hong Kong (29).

To establish the score conversions, we utilized equipercentile linking (30, 31), a method that matches two scales' cumulative distributions and computes equivalent scores from one scale to the other. Log-linear presmoothing of the raw score frequencies was undertaken to reduce random error in the linking process (32). We applied a model selection based on the Bayesian Information Criterion to select the univariate and bivariate models (33), where we considered up to eight univariate moments and three bivariate moments. The model fit was evaluated graphically by comparing the predicted and observed conditional means and variances.

Previous studies have identified a significant association between MoCA sum scores and age and educational level, while item properties of the MoCA varied with education (10, 34, 35). Age- and education-corrected cutoff scores were also proposed to identify people with significant and mild neurocognitive disorders and mild cognitive impairment (35, 36). Therefore, we further evaluated population invariance (37) by estimating linking functions in groups defined by (1) educational level (no formal education, 1–6 years of education, and >6 years of education) and (2) age group (<75 years and ≥75 years). We estimated the linking function from CPS and CPS2 to MoCA 5-min with the R package kequate (38) and obtained equivalent scores and standard errors.

## Results

A total of 4,099 individuals participated in the home and community-based care program.

Values in either MoCA or CPS/CPS2 items were missing for 556 (13.5%) individuals who were excluded from the analysis. The final sample included 3,543 participants with valid data on both the MoCA 5-min and CPS. Two-thirds (66.24%) were female, and the average age was 78.86 years (SD = 8.19). The mean score of MoCA 5-min was 18.51 (SD = 5.93). The mean ADL-H score was 0.28 (SD = 0.91), between independent and supervision required, suggesting the sample's low functional impairment. Less than 4% of the sample had a diagnosis of dementia. The mean CPS and CPS2 scores were 0.65 (SD = 0.69) and 1.34 (1.09), respectively, corresponding to a cognitive performance level between intact and borderline intact. The highest scores observed were 3 for CPS and 5 for CPS2. Table 1 summarizes the mean MoCA 5-min, CPS, and CPS2 scores by age group and educational level. A considerable difference in mean MoCA 5-min scores was evident between people younger than 75 and people aged 75 years or older.

**Table 1:** Sample characteristics.

	**Total Sample**	**<75 years**	**≥75 years**	**No education**	**1–6 years education**	**>6 years education**
	***N* = 3,543**	***N* = 1,074**	***N* = 2,469**	***N* = 1,026**	***N* = 1,489**	***N* = 957**
Age (years) (mean, SD)	78.86 (8.19)	68.70 (3.83)	83.28 (5.04)	79.07 (8.12)	78.86 (8.04)	78.64 (8.49)
Female (%)	66.24	67.69	65.61	68.32	66.25	64.01
ADL-H (mean, SD)	0.28 (0.91)	0.28 (0.94)	0.28 (0.90)	0.29 (0.90)	0.28 (0.92)	0.27 (0.91)
Dementia diagnosis (%)	3.90	2.14	4.66	5.06	3.43	3.37
MoCA 5-min (mean, SD)	18.51 (5.93)	21.16 (5.15)	17.36 (5.87)	18.23 (5.87)	18.49 (5.88)	18.85 (6.05)
CPS (mean, SD)	0.65 (0.69)	0.53 (0.65)	0.70 (0.70)	0.68 (0.70)	0.64 (0.69)	0.63 (0.68)
CPS2 (mean, SD)	1.34 (1.09)	1.13 (1.07)	1.42 (1.08)	1.38 (1.08)	1.33 (1.09)	1.29 (1.09)

**Figure 1:**
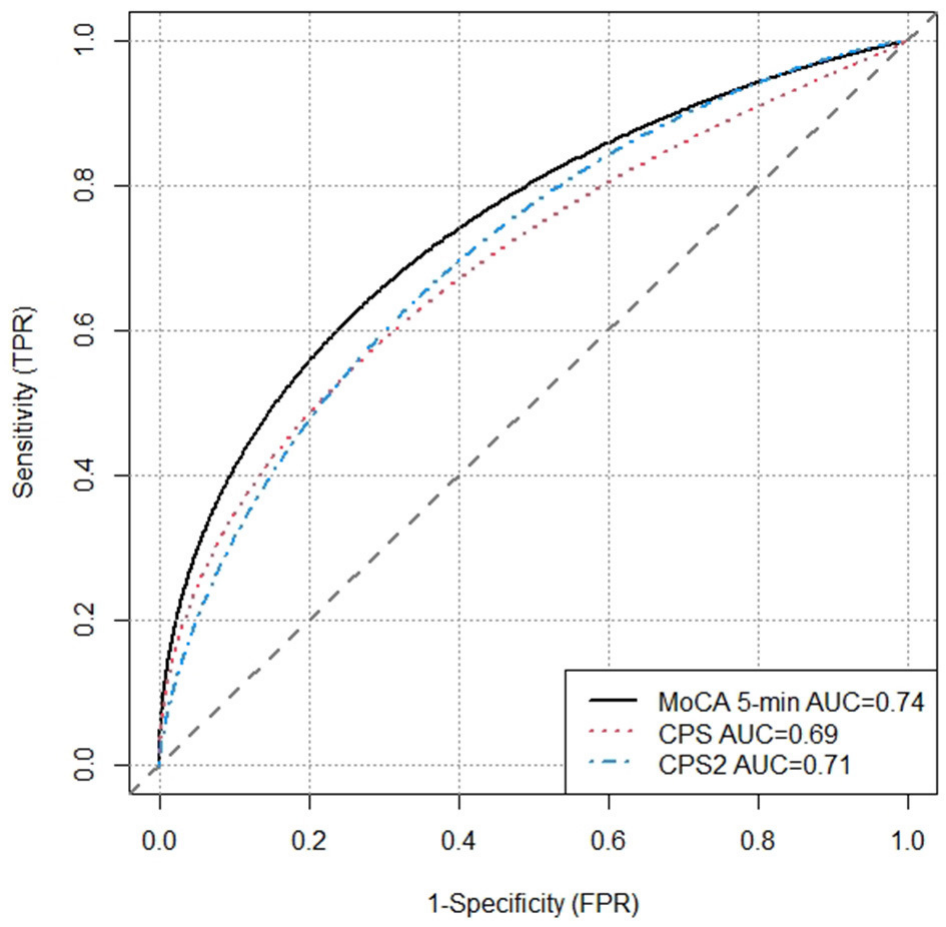
Receiver operating characteristic (ROC) curves for CPS, CPS2, and MoCA 5-min.

The Pearson correlation was −0.42 (*p* < 0.001) between CPS and MoCA 5-min, −0.43 (*p* < 0.001) between CPS2 and MoCA 5-min, and 0.92 (*p* < 0.001) between CPS and CPS2. Kruskal-Wallis tests confirmed significant associations between MoCA 5-min and CPS/CPS2. Table 2 shows that the mean ages, ADL-H, and MoCA 5-min scores differed significantly and substantially by the level of CPS and CPS2. Higher CPS and CPS2 scores are associated with older age, more severe functional impairment as measured by ADL-H, lower MoCA 5-min scores, and higher proportions of people with a dementia diagnosis. We further explored the diagnostic performance of the three scales for dementia using logistic regressions. The receiver operating characteristic curve (ROC) curves are plotted in Figure 1. All three scales detected dementia diagnosis with reasonable accuracy. The area under curve (AUC) was 0.69 for CPS, 0.71 for CPS2, and 0.74 for MoCA 5-min.

**Table 2:** Age, functional assessment scores, MoCA 5-min scores, and dementia diagnosis by level of the CPS and CPS2.

	**CPS score**						**Kruskal-Wallis**
	**0**	**1**	**2**	**3**	**4**	**5**	**test *p*-value**
Sample size	*N* = 1,656	*N* = 1,474	*N* = 395	*N* = 14	-	-	
Age, years	77.64 (8.19)	79.77 (7.93)	80.34 (8.38)	84.50 (8.08)	-	-	0.000
ADL-H	0.22 (0.79)	0.27 (0.90)	0.51 (1.25)	1.93 (2.13)	-	-	0.000
MoCA 5-min	20.83 (4.96)	17.30 (5.63)	13.66 (6.03)	9.86 (8.22)	-	-	0.000
Dementia diagnosis, *n* (%)	34 (2.1%)	49 (3.3%)	51 (12.9%)	3 (21.4%)	-	-	0.000
	**CPS2 score**						
	**0**	**1**	**2**	**3**	**4**	**5**	
Sample size	*N* = 1,138	*N* = 518	*N* = 1,545	*N* = 244	*N* = 85	*N* = 9	
Age, years	77.07 (7.84)	78.87 (8.81)	79.78 (7.92)	80.58 (8.61)	80.25 (8.18)	83.89 (9.79)	0.000
ADL-H	0.12 (0.57)	0.45 (1.09)	0.29 (0.93)	0.56 (1.33)	0.38 (0.95)	1.89 (2.26)	0.000
MoCA 5-min	21.60 (4.72)	19.15 (5.06)	17.17 (5.67)	13.76 (6.05)	12.38 (6.21)	11.33 (9.25)	0.000
Dementia diagnosis, *n* (%)	11 (1.0%)	23 (4.4%)	56 (3.6%)	32 (13.1%)	13 (15.3%)	2 (22.2%)	0.000

For the log-linear models used in the equipercentile linking with the full sample, we selected models with five univariate moments for the MoCA 5-min, two univariate moments for the CPS and four univariate moments for the CPS2. Meanwhile, both selected bivariate models (one for CPS and MoCA 5-min and one for CPS2 and MoCA 5-min) had one bivariate moment. In a single group linking design, we used equipercentile linking in the kernel equating framework with a uniform kernel (31, 39). This approach provides linking functions that closely match traditional linking with percentile ranks while enabling the estimation of random linking error. We selected models that included between four and six univariate moments for the MoCA 5-min for the education- and age-based sub-analyses. All selected models had two and four univariate moments for the CPS and CPS2, respectively, and one bivariate moment for all cases considered. Supplementary Table 1 provides detailed descriptions of the selected models. The relationships between each MoCA score and the conditional mean and variance of the CPS/CPS2 score for the total sample and subgroups are plotted in Supplementary Figures 1–12. Model evaluation based on the fitted and observed conditional means and variances indicated acceptable fit for all models. Discrepancies between the fitted and observed values were only observed at the highest and lowest values of MoCA, which can be expected due to the small number of observations available at the extremes.

**Figure 2:**
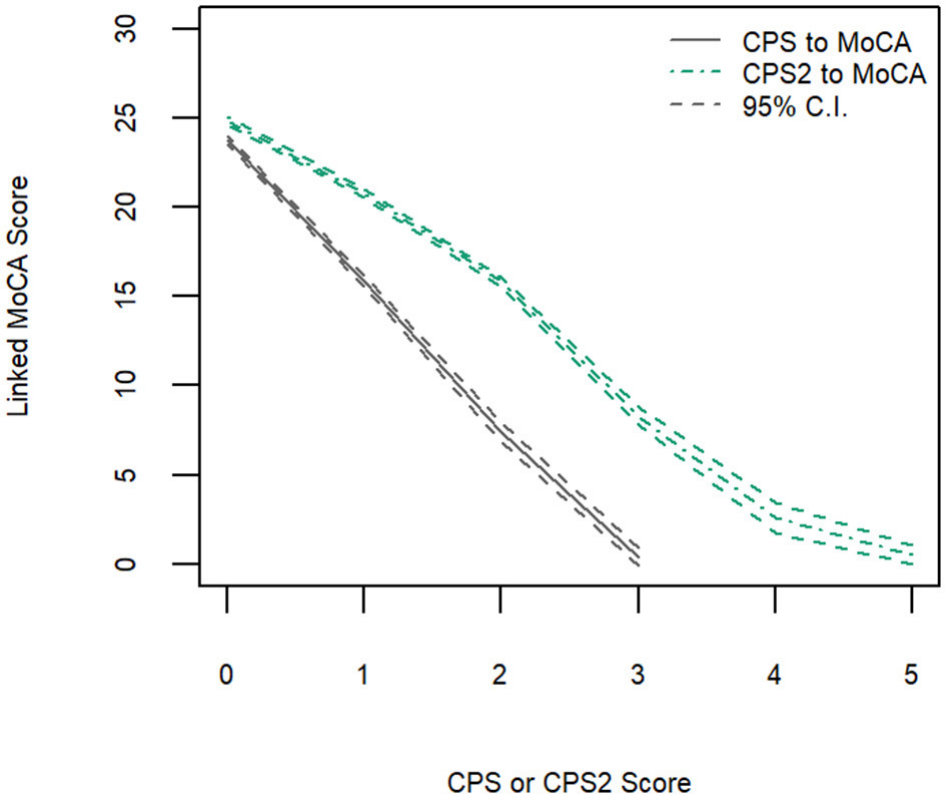
Equipercentile linking function from CPS and CPS2 to MoCA-5 min with 95% confidence intervals.

Figure 2 shows the linking functions from CPS and CPS2 to MoCA 5-min for the full sample, where an approximately linear function existed for the CPS to MoCA 5-min conversion but not for the CPS2 to MoCA 5-min conversion. The random error, low in general, was larger for higher CPS and CPS2 score values, reflecting the lower number of participants with high CPS and CPS2 scores.

**Figure 3:**
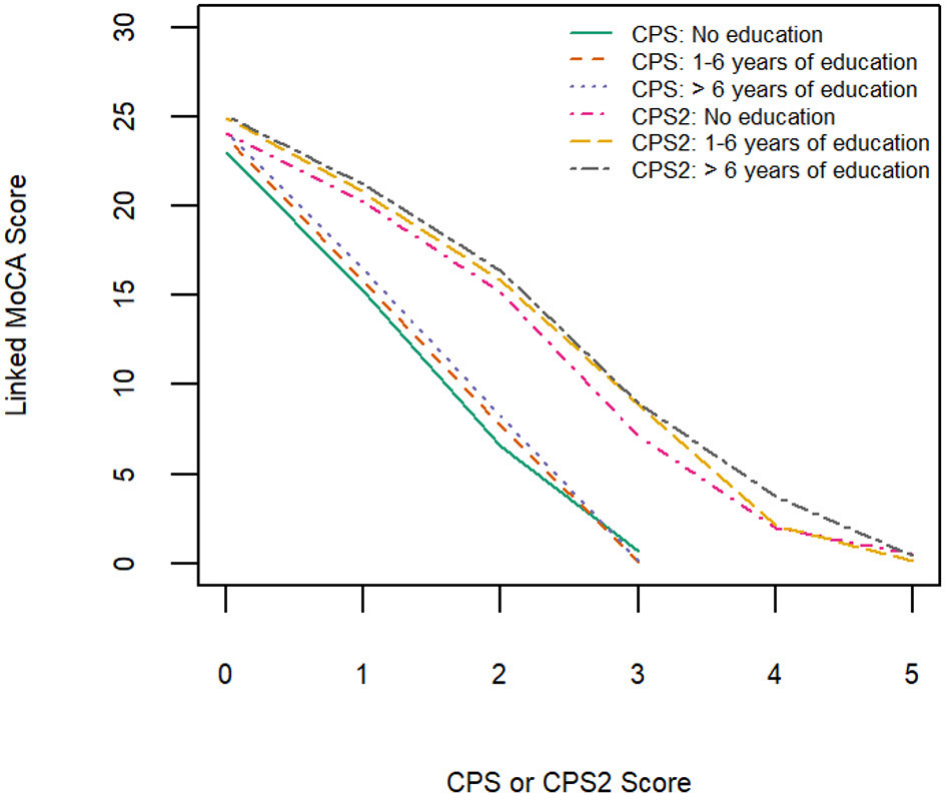
Equipercentile linking function from CPS and CPS2 to MoCA-5 min in three education groups.

Table 3 shows equivalent MoCA scores for each CPS and CPS2 score, for the total sample and by age and education groups. The scores displayed are rounded values to facilitate direct clinical usage. Supplementary Table 2 documents the more accurate estimates of the equivalent MoCA scores and their confidence intervals. The total sample analysis implies that a CPS of 0 corresponds to a MoCA 5-min score of 23.8 (95% CI, 23.5–24.0), and a CPS2 score of 0 corresponds to a MoCA 5-min score of 24.8 (24.6–25.0). At the higher end, a CPS score of 3 and a CPS2 score of 5, the highest scores observed for each of the scales in the sample, correspond to MoCA 5-min scores approximately equal to 0.4 (−0.08 to 0.89) and 0.6 (0.01–1.10), respectively. The linking functions revealed the floor and ceiling effects for the different scales, with CPS and CPS2 capable of measuring more severe cases of cognitive impairment than MoCA 5-min while the MoCA 5-min can measure less severe impairment more accurately than CPS and CPS2. Figure 3 shows that the score conversions did not differ by age groups but differed by educational levels. Participants with no formal education had lower linked MoCA 5-min scores than their counterparts with higher levels of education, although the differences were not substantial. The largest difference (1.87) was observed in linked MoCA 5-min scores between the no education group and >6 years education group.

**Table 3:** Equivalent MoCA scores for each CPS and CPS2 score in three education groups and two age groups.

	**Total Sample**	**<75 years**	**≥75 years**	**No education**	**1–6 years education**	**>6 years education**
*CPS score*						
0 Intact	24	24	24	23	24	24
1 Borderline intact	16	16	16	15	16	16
2 Mild impairment	7	7	8	7	8	8
3 Moderate impairment	0	1	1	1	0	0
*CPS2 score*						
0 Intact 1	25	25	25	24	25	25
1 Intact 2	21	21	21	20	21	21
2 Borderline intact 1	16	16	16	15	16	16
3 Borderline intact 2	8	8	8	7	9	9
4 Moderately impaired 1	3	3	3	2	2	4
5 Moderately impaired 2	1	1	1	1	0	0

## Discussion

This study provides score conversions between MoCA 5-min and CPS/CPS2 in a large cohort of Hong Kong older adults with mild physical or cognitive impairment. This crosswalk enabled uninterrupted assessments of cognitive performance and record-linkage across care settings. We also provide score conversions specific to various age groups and educational levels when more fine-grained conversion is preferred for the study population.

Score conversions among various cognitive assessment tools have been made available to (1) enable continuity in repeated assessments with different tools, (2) improve comparability of cognitive scores generated from different measures in different populations and research settings, and/or (3) facilitate the adoption of newly proposed assessment tools. For example, one study used clinical cohorts with and without neurologic conditions to bridge scores of the short MoCA (s-MoCA) and MMSE (40). A Hong Kong study converted MMSE scores to both MoCA and MoCA 5-min using another clinical sample of patients with stroke or TIA (15). However, previous work is limited to assessment tools within the clinical setting. Large-scale cognitive assessments performed in long-term care settings have long been treated as an independent domain although many people living with dementia receive care in the long-term care system. From a person-centered perspective, longitudinal records that can be viewed, understood, and compared irrespective of care setting are important to ensure the quality of care while controlling the cost. Linking scores of widely adopted tools in healthcare and social care settings are hence needed.

We recruited a sample of older adults who applied to a pilot home care and support for elderly persons with mild impairment program. People with severe cognitive impairment were excluded because of their eligibility for other subsidized services such as enhanced home and community care services and integrated home care services. Consequently, we observed a ceiling effect of CPS and CPS2 attributable to the study sample's unique characteristics. Score conversions between the two scales derived from this study were hence limited to lower values of CPS. However, it is arguable that this range of CPS scores is the most relevant score interval that requires linking as it represents a critical transitional period from independent to needing care or from community to long-term care facilities. Specifically, when MoCA tests have been performed before a nursing home placement, score conversions can be used to understand whether a substantial change in cognition occurred before and after the placement. This may further aid the development of a more personalized care plan or intervention. Alternatively, a nursing home resident or a person receiving home care may also receive a MoCA assessment outside the long-term care setting. Then, placing the MoCA and CPS scores on a common scale enables more frequent monitoring of the trajectory of cognitive decline, which may help detect subtle changes and changes occurring over a short time. For people in long-term care with a moderate to severe level of cognitive impairment, the MoCA may not have been used in the first place as it was designed to screen for mild cognitive impairment and because floor effects of MoCA items have been reported in previous studies (15, 41). Future work on bridging MMSE scores to CPS and CPS2 scores are needed to enable the continued assessment of cognitive performance in people with severe cognitive impairment.

Score conversions between MoCA 5-min and CPS can also benefit research that aims to estimate the monetary cost and societal impact of dementia. These kinds of cost-of-illness studies typically aim to estimate total costs of care for all people with cognitive impairment or dementia in three categories: health, social, and unpaid care. All three cost categories need to be estimated separately by the severity of cognitive impairment or dementia (e.g., mild, moderate, and severe) and then be summed together, which requires a consistent measure to approximate the severity (42).

This study is also the first to explore the criterion validity of CPS in a mild impairment population in an Asian society. The criterion validity was explored using the ROC analysis, and the large AUC values demonstrated that the interRAI CPS and CPS2 can distinguish dementia diagnosis well.

We found differences in linking functions between people with different educational levels. This may be explained by possible differences in characteristics of specific MoCA 5-min items in people with diverse educational backgrounds. An earlier Hong Kong study of MoCA found that the functioning of some items was superior in people without formal education (34). Item-level analysis of the MoCA 5-min was beyond the scope of this investigation. However, future studies are needed to investigate measurement invariance of MoCA 5-min items in more diverse subpopulations.

**Figure 4:**
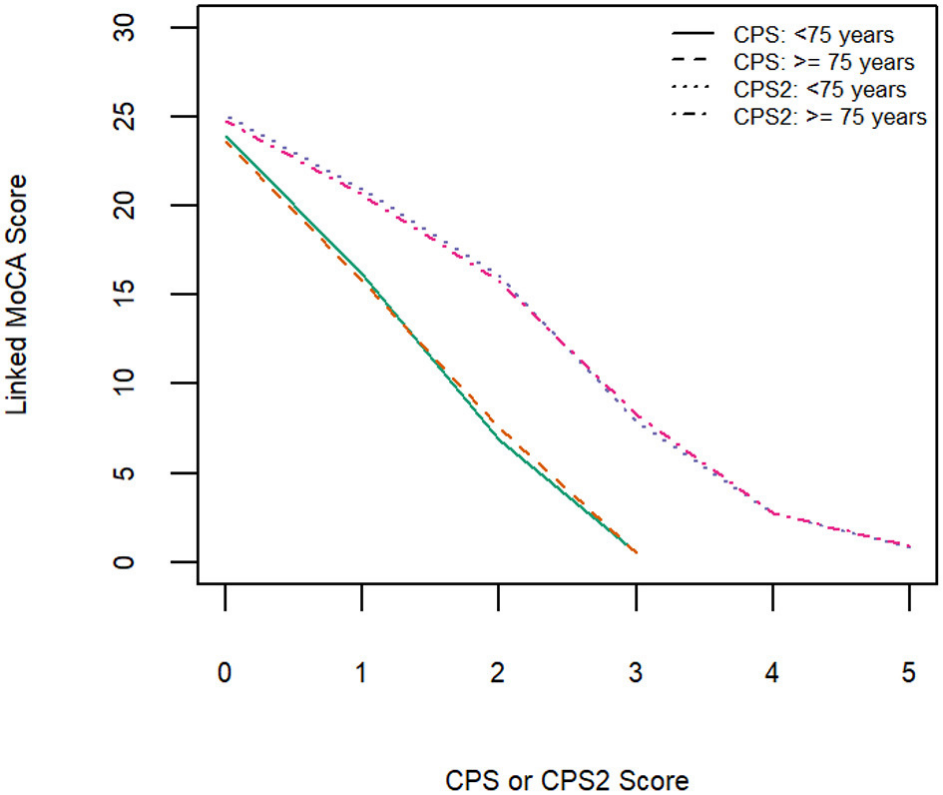
Equipercentile linking function from CPS and CPS2 to MoCA-5 min in two age groups (<75 years old and ≥75 years old).

This study has several limitations. First, our data were collected from community-dwelling older adults with mild functional impairment, excluding people with severe cognitive impairment. Consequently, our highest observed scores were 3 for CPS and 5 for CPS2 scores. Linked MoCA 5-min scores for CPS scores of 4 (moderate for severe impairment) and above, and CPS2 scores of 6 (severe impairment Level 1) and above could not be estimated. However, our results showed that the linked MoCA 5-min score for a CPS score of 3 was already as low as 0, suggesting that higher CPS scores may correspond to a MoCA 5-min score of 0 or a missing value due to severe impairment. Second, although interRAI assessments collect information on the diagnosis of Alzheimer's disease and dementia, only 4% of the participants reported having a diagnosis of dementia, suggesting that dementia is under-diagnosed in community samples in Hong Kong. We hence did not further explore the classification accuracy of the linked scores of MoCA 5-min. The results of the ROC analysis should also be interpreted with caution. Third, the Pearson correlation coefficients between MoCA 5-min and CPS/CPS2 were relatively low. Possible explanations for this are that (1) the CPS/CPS2 suffered from a ceiling effect and only a few values were available for estimation; (2) both CPS/CPS2 and MoCA 5-min are subject to measurement errors that weaken the correlation observed, and (3) the relationships between CPS/CPS2 and MoCA 5-min were not strictly linear as shown in Figures 2–4. It is worth noting here that we aimed to achieve comparability of scores obtained from the two scales using linking rather than equating. Linking can be conducted when two distinct tests measure similar constructs for a common population, while equating requires the more specific condition that tests measure the same (not similar) construct and have equal reliability. To conduct a linking, a high correlation is preferred but not required (43). Fourth, our data were collected from applicants for public-funded home care services in Hong Kong who possibly had lower socioeconomic status. The results may not generalize to other populations.

## Conclusions and Implications

This study represents one of the first attempts to bridge scores generated from cognitive assessment tools commonly used in clinical populations and among older adults in the long-term care system. Cross-sectionally, it bridges scores from cognitive scales used in diverse settings and different research cohorts. Longitudinally, it allows continuous tracking of cognitive performances across the continuum of care. Subject to the unique characteristics of the study sample, score conversions were limited to CPS scores between 0 tand 3, CPS2 scores between 0 and 5, and MoCA 5-min scores between 0 and 24, corresponding to older adults who were cognitively intact or had mild cognitive impairment. Future research bridging scores from a wider range of cognitive assessment tools is warranted to realize continuous tracking of cognitive performance across the continuum of care.

## Data Availability Statement

The datasets presented in this article are not readily available because the ethical approvals do not allow sharing of raw data of this project. De-identified data may be shared separately with qualifying researchers after reviewing the research proposal. The proposal needs to comply with legislation and within the scope of the ethical approval. Requests to access the datasets should be directed to Terry Y. S. Lum, tlum@hku.hk.

## Ethics Statement

The studies involving human participants were reviewed and approved by the Review Board of the Human Research Ethics Committee for Non-Clinical Faculties at the University of Hong Kong (EA1709028). The patients/participants provided their written informed consent to participate in this study.

## Author Contributions

HL, BA, GW, and TL: study concept and design and critical revision of the manuscript for important intellectual content. GW and TL: acquisition of data. BA: analysis and interpretation of data. HL: conducted the analysis and all authors contributed to the interpretation of data. HL and BA: drafting of the manuscript. All authors contributed to the article and approved the submitted version.

## Funding

This work was supported by the Research Grants Council of Hong Kong (grant numbers R7017-18).

## Conflict of Interest

The authors declare that the research was conducted in the absence of any commercial or financial relationships that could be construed as a potential conflict of interest.

## Publisher's Note

All claims expressed in this article are solely those of the authors and do not necessarily represent those of their affiliated organizations, or those of the publisher, the editors and the reviewers. Any product that may be evaluated in this article, or claim that may be made by its manufacturer, is not guaranteed or endorsed by the publisher.
