# Supplementary material for: Linking the Scores of the Montreal Cognitive Assessment 5-min and the interRAI Cognitive Performance Scale in Older Adults With Mild Physical or Cognitive Impairment
Source: Front Psychiatry. 2021 Sep 14;12:705188. doi: 10.3389/fpsyt.2021.705188 (PMC8477039; doi:10.3389/fpsyt.2021.705188)
Supplement: Supplementary file 1 [file Data_Sheet_1.PDF]

Supplementary material

Table 1: Number of moments and cross-moments for selected log-linear model for the total sample and sub-samples stratified by age and by educational level

	MoCA	CPS	CPS2	MoCA × CPS	MoCA × CPS2
Full sample	5	2	4	1	1
No Edu	4	2	4	1	1
Low Edu	5	2	4	1	1
High Edu	6	2	4	1	1
$< 75 years$	4	2	4	1	1
$>= 75 years$	6	2	4	1	1

Table 2: Equivalent MoCA scores for each CPS and CPS2 score in three education groups and two age groups.

	$e^{\text{Total Sample}}_{\text{MoCA 5-min}}$	$e^{\text{NO EDU}}_{\text{MoCA 5-min}}$	$e^{\text{1-6 years EDU}}_{\text{MoCA 5-min}}$	$e^{\text{6+ years EDU}}_{\text{MoCA 5-min}}$	$e^{< 75 years old}_{\text{MoCA 5-min}}$	$e^{\geq\text{75 years old}}_{\text{MoCA 5-min}}$
**CPS Score**						
0 Intact	23.75 (23.54, 23.96)	22.97 (22.58, 23.36)	23.80 (23.47, 24.13)	24.13 (23.79, 24.48)	23.88 (23.53, 24.24)	23.64 (23.37, 23.90)
1 Borderline intact	15.85 (15.57, 16.14)	15.25 (14.70, 15.80)	15.81 (15.39, 16.23)	16.48 (15.85, 17.11)	16.13 (15.60, 16.66)	15.79 (15.46, 16.13)
2 Mild impairment	7.43 (6.87, 8.00)	6.56 (5.81, 7.31)	7.74 (6.98, 8.51)	8.24 (7.25, 9.22)	6.85 (5.89, 7.82)	7.55 (7.03, 8.07)
3 Moderate impairment	0.40 (-0.08, 0.89)	0.67 (0.03, 1.31)	0.10 (-0.37, 0.57)	0.17 (-0.47, 0.80)	0.51 (-0.38, 1.40)	0.53 (-0.03, 1.09)
**CPS2 Score**						
0 Intact 1	24.82 (24.61, 25.04)	24.08 (23.69, 24.48)	24.93 (24.62, 25.25)	25.04 (24.69, 25.39)	25.04 (24.69, 25.40)	24.76 (24.49, 25.03)
1 Intact 2	20.76 (20.51, 21.01)	20.20 (19.73, 20.67)	20.74 (20.35, 21.13)	21.23 (20.77, 21.68)	20.93 (20.50, 21.35)	20.62 (20.30, 20.94)
2 Borderline intact 1	15.84 (15.55, 16.12)	15.16 (14.61, 15.71)	15.89 (15.47, 16.32)	16.41 (15.78, 17.04)	16.11 (15.58, 16.63)	15.79 (15.46, 16.11)
3 Borderline intact 2	8.28 (7.77, 8.79)	7.12 (6.30, 7.94)	8.87 (8.17, 9.56)	8.99 (8.14, 9.84)	7.89 (6.96, 8.81)	8.27 (7.64, 8.89)
4 Moderately impaired 1	2.59 (1.75, 3.44)	2.01 (0.75, 3.28)	2.14 (0.35, 3.94)	3.74 (1.46, 6.03)	2.78 (1.43, 4.12)	2.72 (1.87, 3.57)
5 Moderately impaired 2	0.56 (0.01, 1.10)	0.52 (-0.17, 1.22)	0.15 (-0.46, 0.76)	0.47 (-0.52, 1.47)	0.83 (-0.12, 1.77)	0.90 (0.14, 1.67)

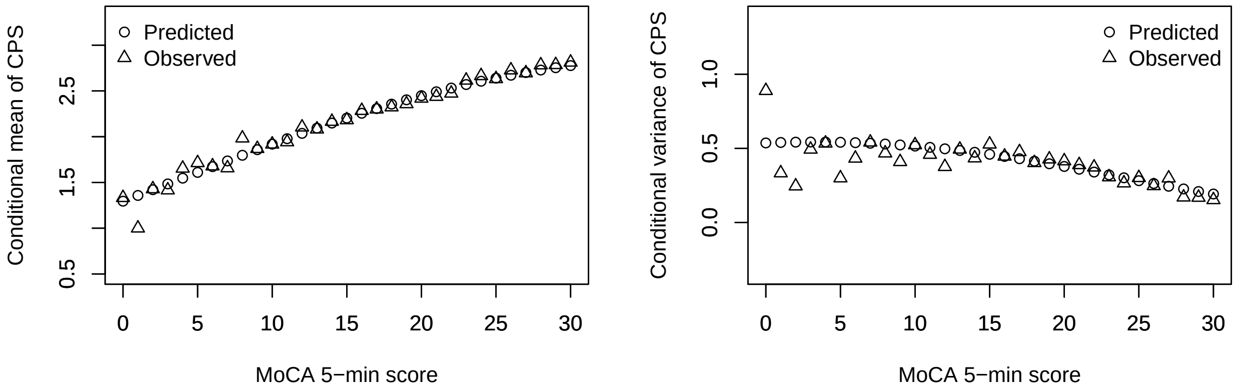

Figure 1: Fitted and observed conditional means and variances of CPS scores (reverse coded) for each value of MoCA

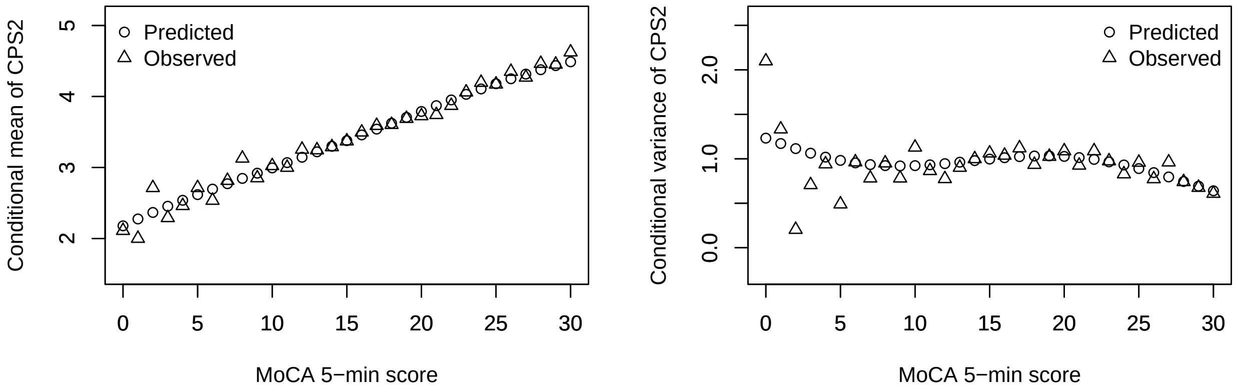

Figure 2: Fitted and observed conditional means and variances of CPS2 scores (reverse coded) for each value of MoCA

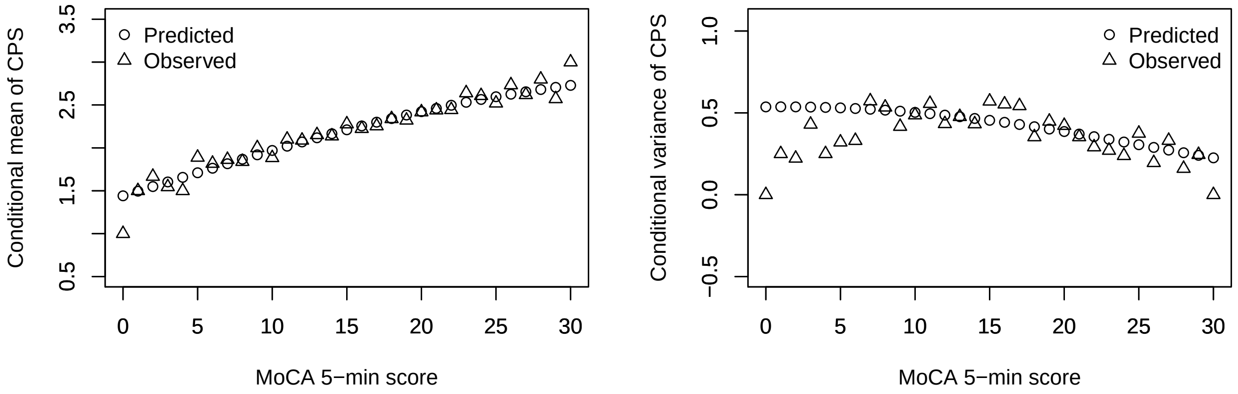

Figure 3: Fitted and observed conditional means and variances of CPS scores (reverse coded) for each value of MoCA in people without formal education

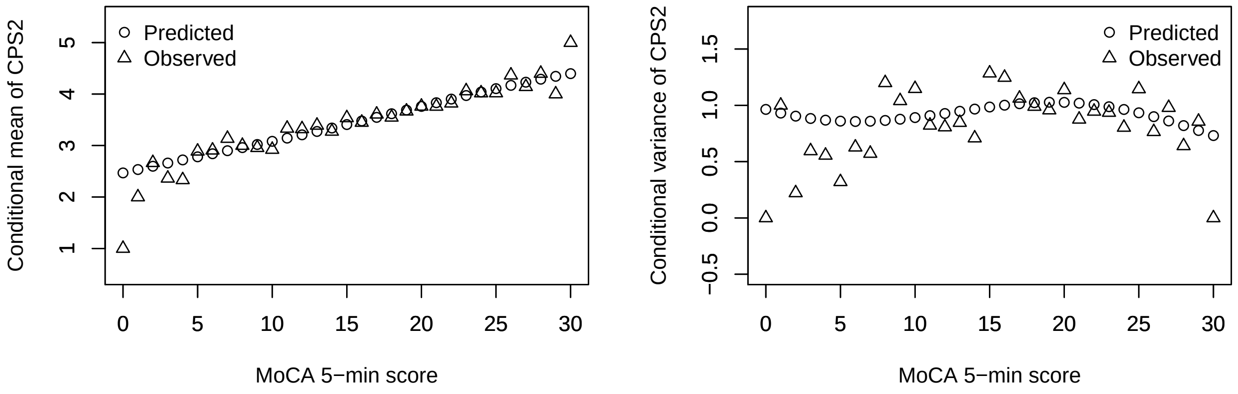

Figure 4: Fitted and observed conditional means and variances of CPS2 scores (reverse coded) for each value of MoCA in people without formal education

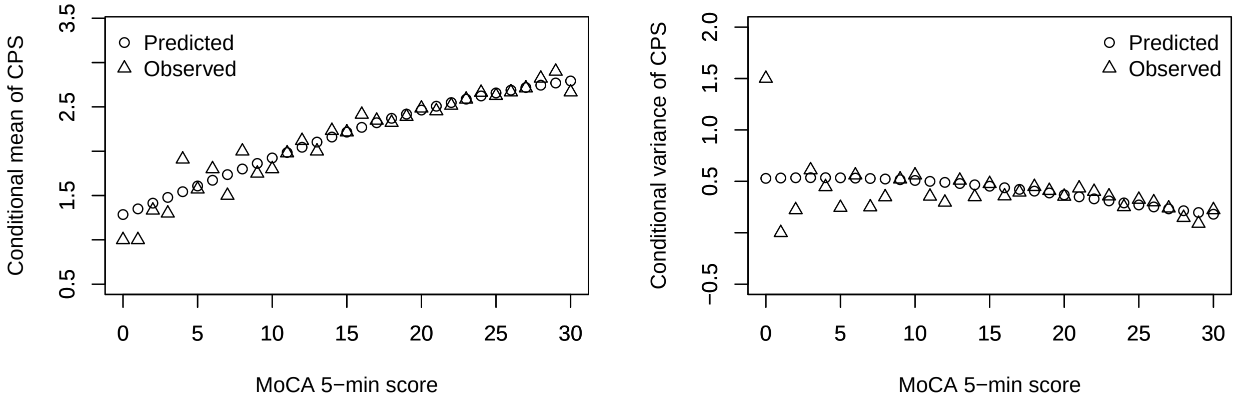

Figure 5: Fitted and observed conditional means and variances of CPS scores (reverse coded) for each value of MoCA in people with low education

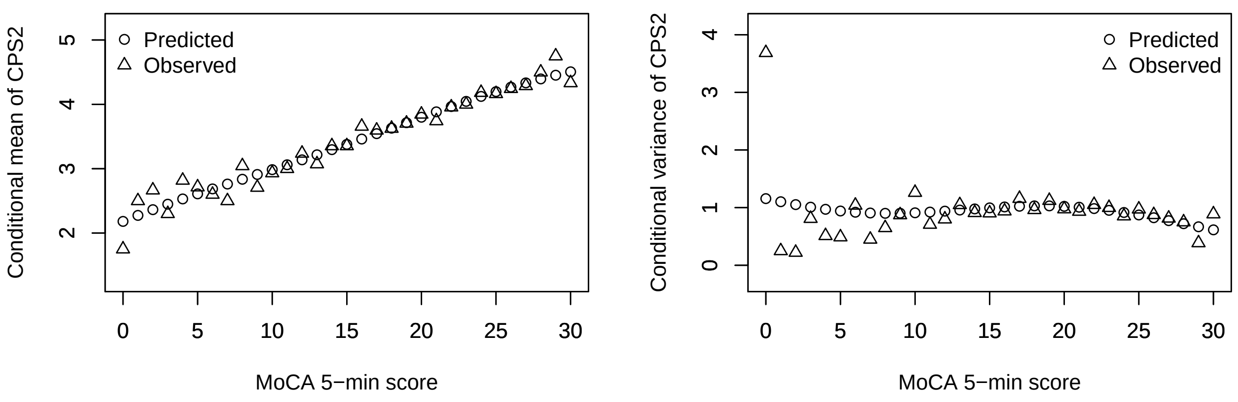

Figure 6: Fitted and observed conditional means and variances of CPS2 scores (reverse coded) for each value of MoCA in people with low education

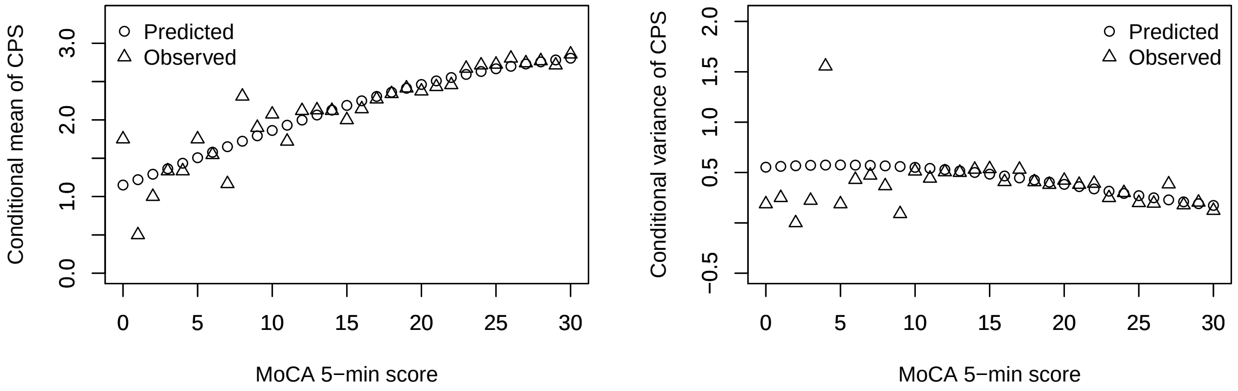

Figure 7: Fitted and observed conditional means and variances of CPS scores (reverse coded) for each value of MoCA in people with high education

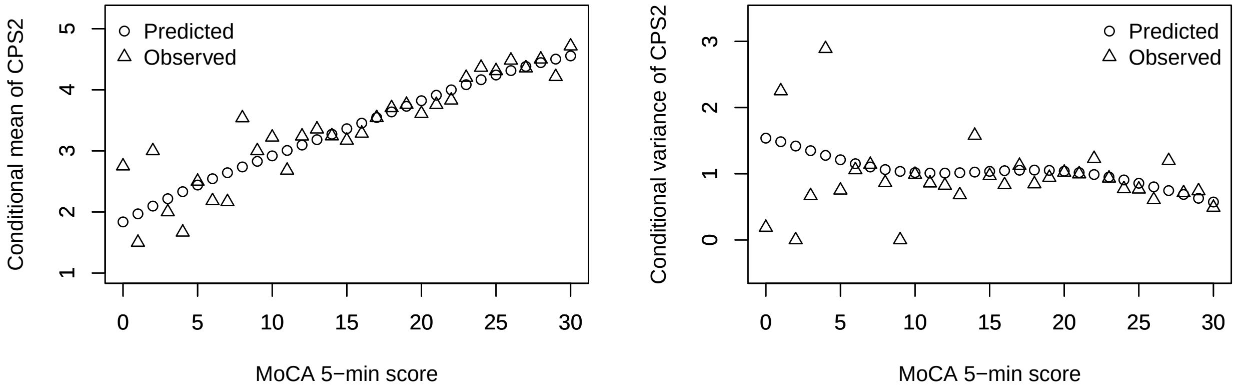

Figure 8: Fitted and observed conditional means and variances of CPS2 scores (reverse coded) for each value of MoCA in people with high education

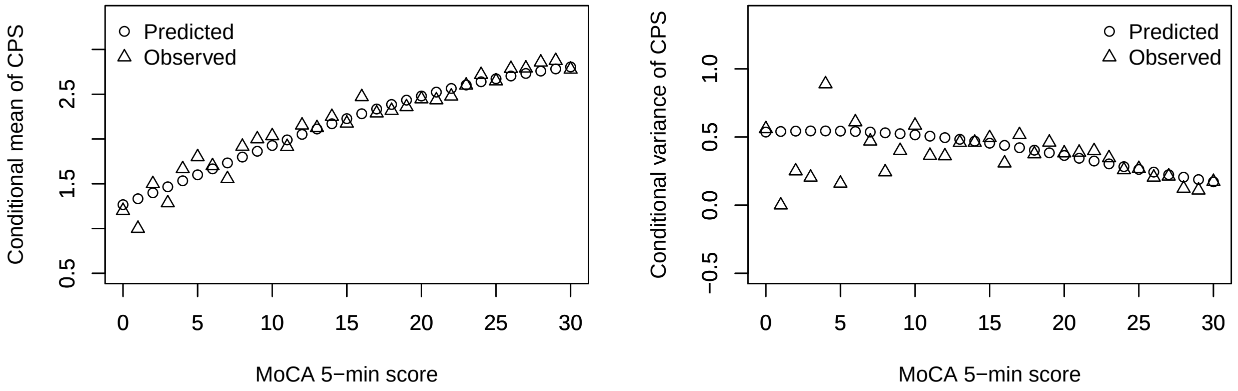

Figure 9: Fitted and observed conditional means and variances of CPS scores (reverse coded) for each value of MoCA in people < 75 years old

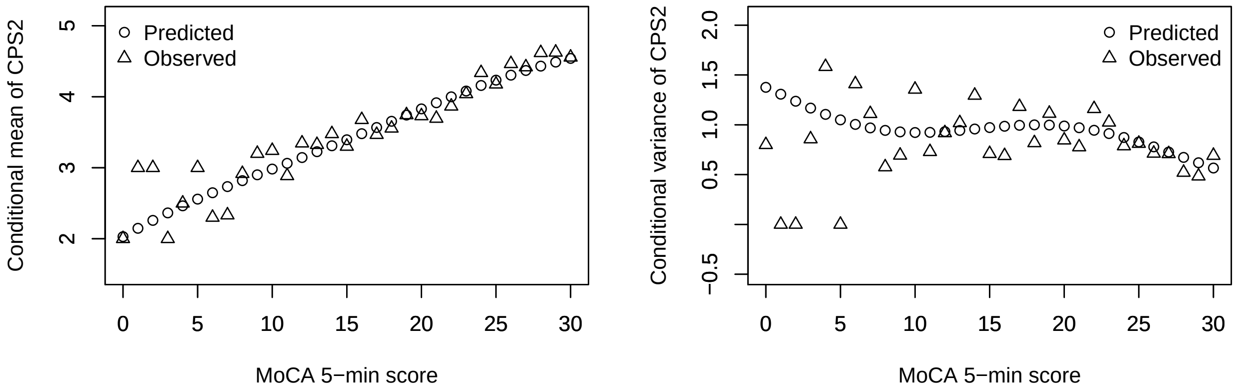

Figure 10: Fitted and observed conditional means and variances of CPS2 scores (reverse coded) for each value of MoCA in people < 75 years old

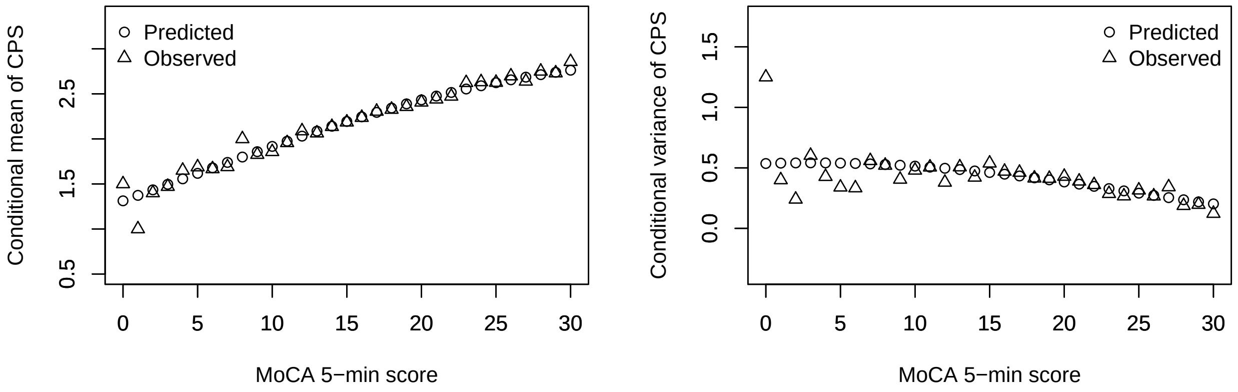

Figure 11: Fitted and observed conditional means and variances of CPS scores (reverse coded) for each value of MoCA in people >= 75 years old

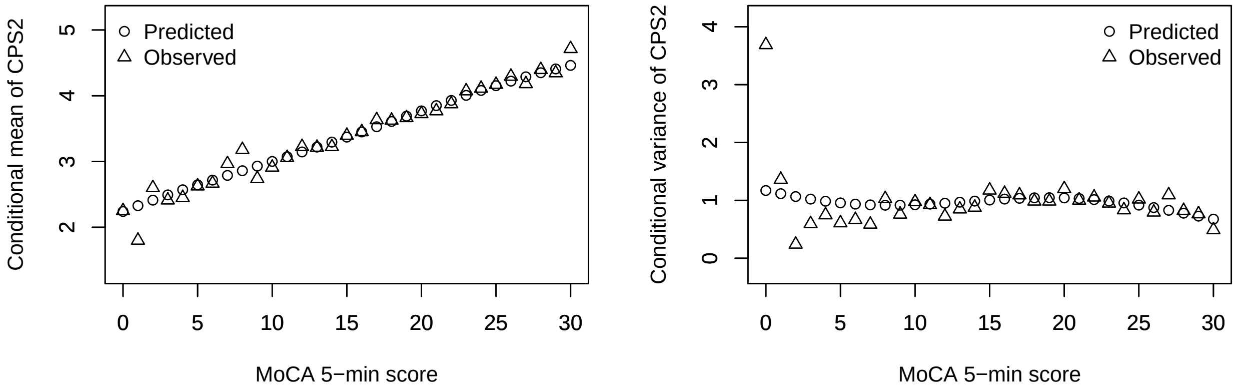

Figure 12: Fitted and observed conditional means and variances of CPS2 scores (reverse coded) for each value of MoCA in people >= 75 years old
